# Small molecule allosteric uncoupling of microtubule depolymerase activity from motility in human Kinesin-5 during mitotic spindle assembly

**DOI:** 10.1038/s41598-019-56173-9

**Published:** 2019-12-27

**Authors:** Catherine D. Kim, Elizabeth D. Kim, Liqiong Liu, Rebecca S. Buckley, Sreeja Parameswaran, Sunyoung Kim, Edward J. Wojcik

**Affiliations:** Department of Biochemistry and Molecular Biology, LSU School of Medicine & Health Sciences Center, 1901 Perdido Street, New Orleans, LA 70112 USA

**Keywords:** Enzymes, Mitotic spindle, Small molecules, Molecular medicine

## Abstract

Human Kinesin-5 (Eg5) has a large number of known allosteric inhibitors that disrupt its mitotic function. Small-molecule inhibitors of Eg5 are candidate anti-cancer agents and important probes for understanding the cellular function. Here we show that Eg5 is capable of more than one type of microtubule interaction, and these activities can be controlled by allosteric agents. While both monastrol and S-trityl-L-cysteine inhibit Eg5 motility, our data reveal an unexpected ability of these loop5 targeting inhibitors to differentially control a novel Eg5 microtubule depolymerizing activity. Remarkably, small molecule loop5 effectors are able to independently modulate discrete functional interactions between the motor and microtubule track. We establish that motility can be uncoupled from the microtubule depolymerase activity and argue that loop5-targeting inhibitors of Kinesin-5 should not all be considered functionally synonymous. Also, the depolymerizing activity of the motor does not contribute to the genesis of monopolar spindles during allosteric inhibition of motility, but instead reveals a new function. We propose that, in addition to its canonical role in participating in the construction of the three-dimensional mitotic spindle structure, Eg5 also plays a distinct role in regulating the dynamics of individual microtubules, and thereby impacts the density of the mitotic spindle.

## Introduction

**Figure 1:**
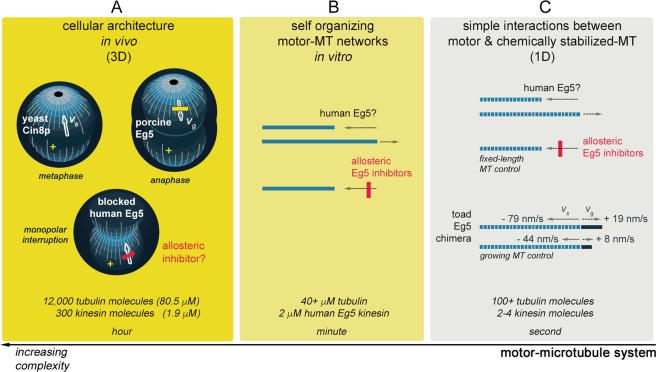
Study of Kinesin-5 regulation of MT dynamics has focused on cellular changes *in vivo* and measurements of individual MT *in vitro*. Effects of kinesin proteins on the microtubule cytoskeleton are studied at different levels, such as (**A**) knockout of Kinesin-5 in cells^6,10,11^, (**B**) self-organizing microtubule-motor systems evaluated by turbidity^27^, and (**C**) analysis of individual microtubules that are either favored to have net MT growth^11^ or fixed in length and not subject to growth and shrinkage^27^. The scale and complexity of the system under study multiplies from right to left in the diagram: protein number and concentration of the players involved increases with each level of experimental measurement. Absolute quantitation of kinesin and tubulin molecules, as well as concentrations, are obtained from eukaryotic cells using two orthogonal methods^2^. In addition, the timescale of measurement deviates by 100-fold between single-molecule measurements and cellular events. In (**B**,**C**) blue bars represent microtubules, blue bars with white stripes highlight use of chemical stabilization of filaments, and dark blue bars represent net elongation of microtubules. Abbreviations: v_s_ = rate of microtubule shrinkage; v_g_ = rate of microtubule growth.

The mitotic spindle is the central microtubule-based scaffold for the segregation of chromosomes to daughter cells during cell division. The dramatic series of spindle rearrangements during mitosis require alteration of tubulin-microtubule equilibrium by molecular motors and other microtubule-associated proteins. *In vitro* identification of proteins that modulate organization of the microtubular spindle has been rising in number. Equally important, there is an increased quantitative understanding of the metaphase spindle size, composition, and lifetime in cells. Examples include: the mitotic spindle size has a linear relationship with the cell volume^1^; concentration of tubulin in animal cells (Fig. 1A) is 80 micromolar^2^; and all microtubules within the spindle have a dynamic turnover every 30 s^3^.

An important challenge is to identify mechanistic links between self-organizing behavior of tubulin and the cellular machines that construct and alter the mitotic spindle. For example, changes in microtubule organization involve Kinesin-5^4^ and dynein, each being ascribed one individual function in spindle morphogenesis: the former is thought to restrict sliding and the latter is involved in spindle elongation. Although Kinesin-5 is one of the earliest mitotic motors identified, there is lack of accordance between cellular and *in vitro* studies aimed at elucidating its function in mitotic spindles. Kinesin-5 proteins form homotetramers, an oligomeric state that suggests that it crosslinks and slides microtubules in an antiparallel fashion^5^. However, Eg5 is detected only to a limited extent at the spindle midzone, where antiparallel microtubules are located, until late anaphase; instead, it is enriched at spindle poles during metaphase and anaphase^6–8^. Thus, models of its mitotic function, based solely on oligomeric organization, are not consistent with its cellular localization.

On the other hand, several Kinesin-5 studies offer evidence that Eg5 may not only slide and stall microtubule gliding but also may modulate microtubule dynamics. Cin8p, the *Saccharomyces* Kinesin-5, has been shown to interact at the plus-ends of kinetochore microtubules near the central midzone and promote depolymerization (Fig. 1A), a mechanism thought to restrict the overall length of microtubules in this cellular system^9^. Experiments in porcine kidney cells^6^ and in *C*. *elegans*^10^ suggested that Kinesin-5 acts as a frictional brake for inappropriate microtubule elongation during mitosis (Fig. 1A). In contrast, an *in vitro* study^11^ determined that addition of purified *Xenopus* Kinesin-5, truncated and in an artificial chimeric form, to growing microtubules would increase both shrinkage rates and growth rates of individual cytoskeletal polymers (Fig. 1). Yet, this chimeric Eg5 primarily promoted net microtubule polymerization *in vitro* in single-molecule assays^11^, a finding at odds with the *in vivo* reports.

This discrepancy between *in vivo* and *in vitro* studies may arise from limitations inherent within each type of investigation. Genetic knockout or physical ablation are key, but blunt, tools to query the function of an individual protein in cells. Studies of kinesin-microtubule interactions *in vitro* have several artificial restrictions for each protein entity. Although the smallest of the cellular nanomotors, the large molecular size of a kinesin protomer and the requirement of oligomerization for cellular movement necessitate protein modification for study. In addition, the dynamics of microtubules often are suppressed by a variety of different stabilizing agents (e.g., non-hydrolyzable analogs of GTP, Taxol, and DMSO) for single particle tracking. Furthermore, the number of molecules and the timescale of the measurements are often at odds between cell and isolated kinesin-microtubule interactions (Fig. 1). An intermediate approach between these two experimental extremes is turbidity, which can measure the effect of motor proteins on self-assembly mechanisms of unmodified tubulin filaments (Fig. 1).

An alternate explanation for the incongruity between *in vivo* and *in vitro* studies is that the working model of Eg5 remains incomplete. Our hypothesis is that Kinesin-5 is not only capable of moving microtubule tracks, but also can modulate microtubule dynamics. This combination of motor-microtubule functions uniquely could reorganize three-dimensional mitotic architecture, size, and density. Human Eg5 is a suitable model for testing if a kinesin has more than one type of functional interaction with microtubules. The ability to modify microtubule length can be uniquely evaluated with existing small-molecule inhibitors of Eg5^12,13^; loop5, which undergoes conformational opening and closing of a pocket during normal catalysis^14^, was initially identified as the binding site for monastrol^15^ and S-trityl-L-cysteine (STC)^16,17^. Application of STC or monastrol to human cells causes the catastrophic failure to assemble the mitotic spindle during cell division, leaving instead a nonfunctional monopolar spindle array^18,19^. Extensive characterization of these inhibitors provides a robust background to utilize these compounds as probes to explore Eg5 function *in vitro* and in dividing human cultured cells. Herein, experiments at the molecular level and at the cellular level consistently support a new finding that human Eg5 has two functions in mitosis: not only is it capable of microtubule-based motility/gliding but also regulates microtubule dynamics. Allosteric inhibitors can affect both of these human Eg5 functions or selectively inhibit only one.

## Results

### The motor domain of wildtype human Eg5 kinesin can cause net depolymerization of microtubules *in vitro*

We set out to determine whether or not human Kinesin-5 could exhibit a microtubule depolymerizing activity *in vitro*, in a manner similar to yeast Cin8p^9^. Initially, our focus was to determine whether human Eg5 can alter *in vitro* microtubule dynamics in turbidity assays at protein concentrations that mirror cellular conditions (Fig. 1A,B). Recently, proteomic efforts have provided *in vivo* quantitative determination of 100+ proteins in a eukaryotic cell^2^; such definitions for intracellular protein concentration are essential for establishing *in vitro* stoichiometry and parameters of motor-microtubule reactions, as well as mathematical modeling of cellular processes. Intracellular tubulin and kinesin concentrations were nearly 80 and 2 μM, respectively (Fig. 1). At such concentrations, cellular self-assembly of tubulin into microtubules is expected even in the absence of stabilizing agents, such as Taxol, GMPCPP, or high DMSO concentrations.

We elected to examine the effect of monomeric, human Eg5 on microtubule shrinkage or growth, as the property of removing tubulin from the cytoskeletal track requires motor domain elements (e.g.^20^), and motility, which requires oligomerization of more than one motor domain (e.g.^21,22^). Eg5 motor domain was purified^14,23^: it can interact with the microtubule lattice, but being an obligate monomer, is incapable of motility^24,25^. Purified tubulin was prepared from bovine brains^26^.

**Figure 2:**
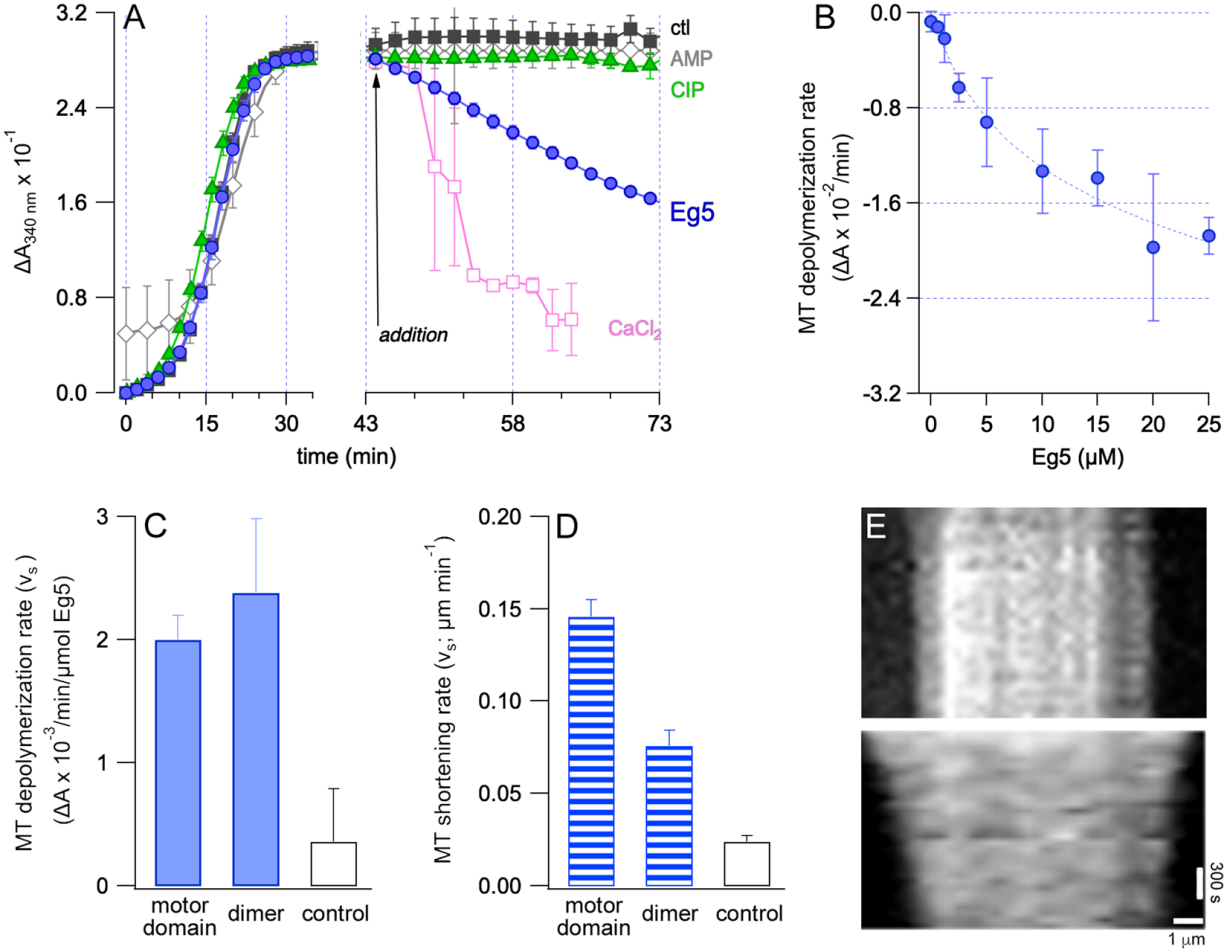
Eg5 promoted microtubule depolymerization *in vitro*. Microtubule shrinkage by human Eg5 were measured by turbidity (panels A–D) and total internal reflection fluorescence microscopy (panels E,F). (**A**) In the left panel, polymerization of 5 mg/mL tubulin was monitored by changes in turbidity at 340 nm as a function of time. These self-assembly reactions occurred in the presence of 1 mM GTP and without stabilizing agents such as Taxol or GMPCPP. In the right panel, changes in tubulin polymerization were examined after additions to the reactions in panel A at 43 min. Reaction additions were vehicle (ctl, black squares); CaCl_2_, a known depolymerizing agent (pink squares); Eg5 motor domain with 1 mM ATP (Eg5, blue circles); Eg5 with 1 mM nonhydrolyzable AMPPNP (AMP, gray diamonds); and Eg5 with calf intestinal phosphatase (CIP, green triangles). (**B**) Eg5 motor domain drives net microtubule depolymerization in an exponential dose-response. We chose 2.5 µM Eg5 for subsequent turbidity experiments. (**C**) In turbidity experiments with 1 mM ATP and 40 µM tubulin, mean (SE) rate of microtubule shrinkage was 2.0 × 10^−3^ (2.0 × 10^−4^) AU/min/µmol Eg5 motor domain, 2.4 × 10^−3^ (6.0 × 10^−4^) AU/min/µmol Eg5-513 (dimer), and 3.5 × 10^−4^ (4.3 × 10^−4^) AU/min for control microtubules (n = 22). (**D**) In TIRF measurements, mean (SE) rate of microtubule shrinkage was 1.5 × 10^−1^ (9.2 × 10^−3^) μm/min, 7.6 × 10^−2^ (8.4 × 10^−3^), and 2.4 × 10^−2^ (3.2 × 10^−3^) μm/min for the Eg5 motor domain (n = 36), Eg5-513, and for control Taxol-stabilized microtubules, respectively (n = 10). (**E**) Representative kymograph of a control microtubule (upper panel) and a microtubule incubated with Eg5 motor domain (lower panel).

In a self-organizing system, without the use of chemical stabilizers, αβ tubulin heterodimers can assemble into microtubule filaments in the presence of GTP if the concentration of tubulin is above a critical concentration. Using four different tubulin concentrations, *in vitro* polymerization was measured as increases in turbidity at 340 nm as a function of time; 40 µM tubulin was the minimum concentration needed for polymerization (Fig. S1A). Turbidity measurement has been shown to be linear if the tubulin polymer is longer than 60 nm^27^. Thus, we established use of 45 µM tubulin for each microwell turbidity assay (Fig. 2A). By polymerizing the microtubules *in situ*, and without any subsequent pelleting or washes, we achieved a uniform suspension that avoided major optical artifacts due to clumping or aggregation of tubulin in our assay (*ctl*, black boxes in Fig. 2A). Microtubules in our assay were readily depolymerized upon addition of CaCl_2_ (pink boxes, Fig. 2A), a known destabilizing agent^28^, indicating only very low levels of stable tubulin aggregates in our assay conditions. GTP-microtubules were stable for many hours at 37 °C (not shown).

*In situ* tubulin polymerization reactions were allowed to achieve a maximum microtubule concentration plateau (Fig. 2A) prior to the addition of different candidate effectors. Upon addition of Eg5 motor domain plus ATP (purple circles, Fig. 2A), we observed marked decreases in turbidity. Decreases in turbidity are a classical readout for microtubule depolymerization^27,29^, and these data suggest that Eg5 is capable of microtubule depolymerization activity. However, although such bulk assays imitate self-organizing motor-MT networks *in vivo*, we cannot exclude that the turbidity decrease by Eg5 may arise from inhibition of polymerization, increased catastrophe rates, or inhibition of nucleation.

The rate of turbidity decrease was dose-dependent on Eg5 concentration, with a rate maxima achieved with nearly 20 µM Eg5 (Fig. 2B). Based on the measured turbidity change with 2.5 µM Eg5, the depolymerization rate for Eg5 motor domain and the dimeric Eg5-513 was calculated as −2 × 10^−3^ and −2.4 × 10^−3^ AU/min/µmol, respectively (Fig. 2C). The latter is a dimeric form of Eg5, previously shown to be capable of plus-end directed motility^30^. In comparison, the control microtubules had a nearly ten-fold lower rate of depolymerization (Fig. 2C), whereas the CaCl_2_ depolymerization rate was ten-fold higher at −1.3 × 10^−2^ AU/min (Fig. 2A).

Since the observed microtubule destabilization involved Eg5 that is actively cycling and hydrolyzing ATP, we asked whether or not ATPase activity was directly coupled to microtubule depolymerization. Based on the kinetic data above, we utilized 2.5 μM Eg5 motor domain to 45 μM microtubules in subsequent turbidity experiments. We find that blocking Eg5 ATPase activity in our turbidity assay by treatment with excess orthosteric inhibitor, AMPPNP, completely inhibited its microtubule depolymerization ability (AMP, grey diamonds in Fig. 2A). The well-characterized rigor binding to the microtubule lattice afforded by Eg5-AMPPNP^31–33^ does not destabilize the lattice.

It is known that, in addition to ATP, the kinesin motor domain is capable of hydrolyzing GTP *in vitro*^34,35^. Therefore, it is conceivable that rapid hydrolysis of GTP by Eg5 may indirectly lead to microtubule depolymerization in our assay. To eliminate this possibility, we tested whether the microtubules in our assay were sensitive to bulk hydrolysis of GTP to GMP + P_*i*_ by addition of alkaline phosphatase, which rapidly hydrolyzes GTP within seconds in our conditions (data not shown). Alkaline phosphatase treatment does not result in measurable microtubule depolymerization in our assay during the duration of our experiments (CIP, green triangles in Fig. 2A). In net, we demonstrate that Eg5 depolymerization is a direct result of the kinesin interaction with the microtubules and not an indirect effect of GTP depletion.

### Eg5 promotes end-associated microtubule depolymerization

In order to characterize the mode of Eg5-mediated microtubule destabilization, we employed an orthogonal assay to observe individual microtubules via TIRF microscopy. Since the TIRF assay requires stable microtubules, Taxol was used to polymerize tubulin at lower tubulin concentrations and at a faster rate (Fig. S1B; such treatment created stable microtubules, which do not undergo steady-state treadmilling and minimize contributions of catastrophe and rescue (control microtubules, Fig. 2D,E)). After preparing Taxol-microtubules with an AlexaFluor555 label for visualization, we diluted samples for TIRF microscopy. Under our assay conditions, the Taxol-stabilized microtubules exhibited a broad size distribution (Fig. S1C) and have negligible rates of shrinkage from microtubule ends (Fig. 2). In contrast, in the presence of Eg5, modest rates of end-mediated microtubule depolymerization was observed (Fig. 2D,E). The rate of microtubule shortening was 0.15 and 0.08 μm/min, for Eg5 motor domain and dimeric Eg5-513, respectively. Control Taxol-stabilized microtubules had a shrinkage rate of 0.02 μm/min. There was no evidence of microtubule elongation or changes in nucleation in the timeframe of our experiments. Motor domain-mediated depolymerization was observed to be asymmetric, with one end of a microtubule exhibiting a more rapid depolymerization (Fig. 2E). These data demonstrated that the human Eg5 *in vitro* is capable depolymerizing microtubules that are self-assembled in near-cellular concentrations and that are stabilized by Taxol.

### Monastrol fails to inhibit the microtubule depolymerizing activity of human Eg5 kinesin

**Figure 3:**
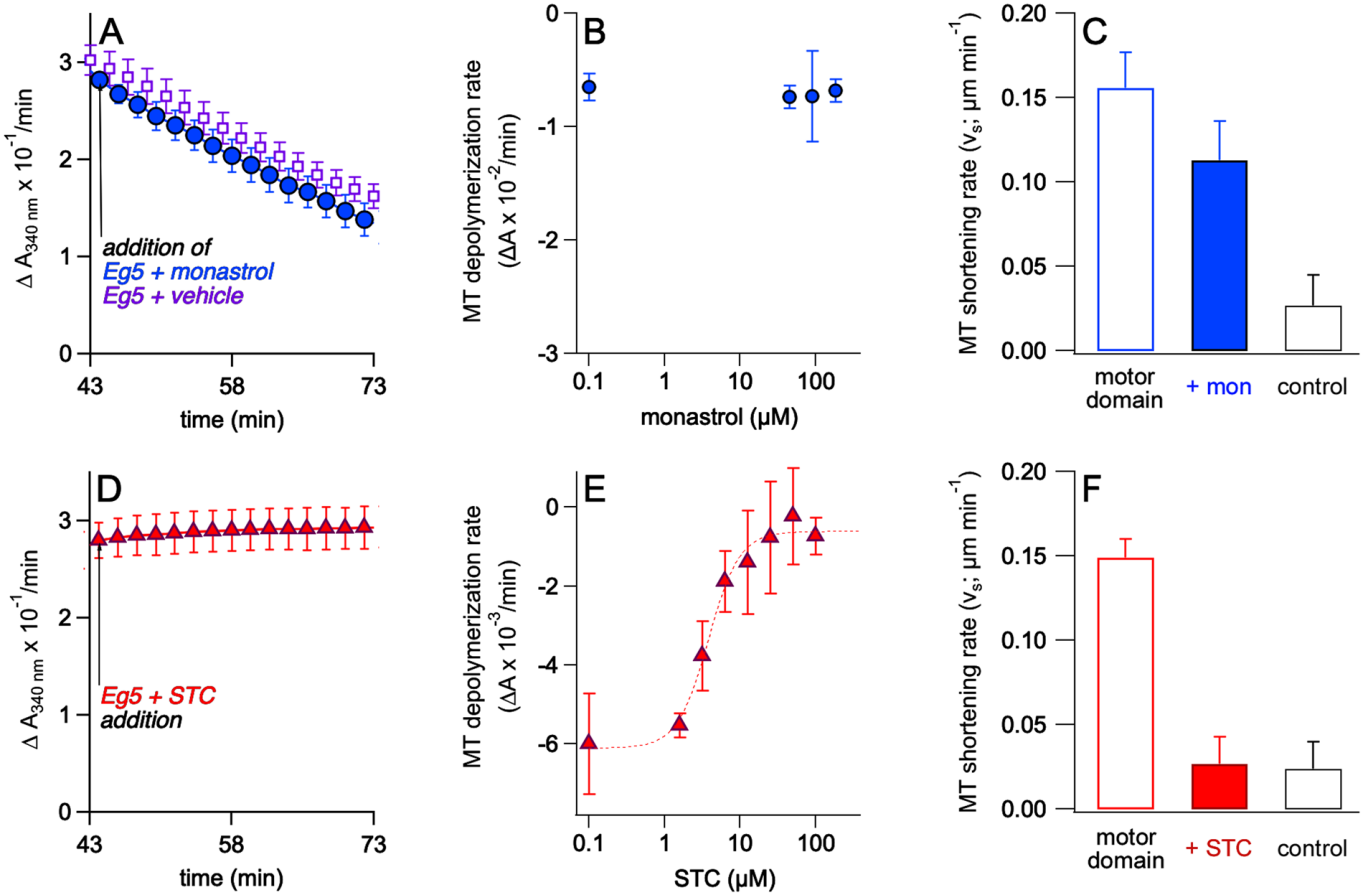
STC treatment inhibited Eg5 microtubule depolymerizing activity, but monastrol does not. (**A**) Either monastrol (closed blue circles, Eg5+ monastrol) or an equivalent volume of DMSO (open blue circles; Eg5+ vehicle) was added concomitantly with Eg5 to polymerized microtubules in turbidity assays. Alteration in rates of microtubule shrinkage by human Eg5 was measured (**B**) by turbidity across a wide range of monastrol concentrations and (**C**) TIRF microscopy microtubule kymograph analysis with Eg5 motor domain treated with 100 μM monastrol. (**D**) Addition of STC (magenta triangles) blocked Eg5-driven changes in turbidity assays. Alteration in rates of microtubule shrinkage by human Eg5 was measured (**E**) by turbidity across a wide range of STC concentrations and (**F**) TIRF microscopy microtubule kymograph analysis of Eg5 motor domain treated with 10 μM STC.

We next explored whether these Kinesin-5 driven changes in microtubule dynamics can be stopped by allosteric inhibitors, recognized for their exquisite selectively and high potency against human Eg5. S-trityl-L-cysteine (STC)^16,19^ and monastrol^18,36^ target the same allosteric pocket formed by the exposed loop5 of the motor domain, and both inhibit Eg5 ATPase activity by blocking the ADP-release step. Neither compound had any direct effect on microtubule stability, in the absence of Eg5, in our assay (not shown). When Eg5 together with molar excess of monastrol were added simultaneously to assembled microtubules, we observed robust microtubule depolymerization in our assay (Eg5+ monastrol, Fig. 3A). The monastrol-treated microtubule depolymerization rate was indistinguishable from that of Eg5 plus vehicle (−2 × 10^−3^ AU/min/µmol). Increasing the dosage of monastrol up to an order of magnitude beyond the reported IC_50_ for inhibition of Eg5 ATPase activity^23,25,37^ did not inhibit Eg5 microtubule depolymerizing activity in either turbidity (Fig. 3B) or TIRF (Fig. 3C) experiments.

### STC inhibits the microtubule depolymerizing activity of human Eg5 *in vitro* and has a depolymerization IC_50_ higher than the median inhibitory concentration for ATP hydrolysis and motility

We next tested STC, a chemically distinct allosteric inhibitor of Eg5 that also targets the loop5 pocket but with atomic contacts that differ from monastrol^17,19,38^. Given that STC blocks product release, we expected a similar impact on Eg5 depolymerizing activity as monastrol, but instead observed the opposite result: STC inhibited the microtubule depolymerizing activity of Eg5 *in vitro* (Fig. 3D). This effect was dosage sensitive and saturable (Fig. 3E), with an IC_50_ of 3.8 ± 0.4 µM; this half maximal inhibitory value is five-fold higher than its inhibition of Eg5 ATPase activity^16,19^ but in reasonable agreement. TIRF measurements (Fig. 3F) also showed that STC was capable of inhibiting Eg5-depolymerizing activity. Given that both monastrol and STC trap the motor in a state that exhibits lower affinity to the microtubule lattice^32,39,40^, it is surprising that this weaker interaction to microtubules promotes microtubule depolymerizing activity. To bolster the turbidity assay as a readout for microtubule depolymerization, microtubule pull-down assays were performed that revealed increasing free tubulin in the presence of monastrol-bound motor, but not STC-bound motor (Fig. S2). Furthermore, Eg5  was found to be roughly equally associated with GTP-stabilized microtubules after treatment with monastrol or STC (Fig. S2). While both drugs strongly inhibit the ATPase activity of the motor, only one compound, STC, can exert small-molecule control of Eg5 microtubule depolymerizing activity *in vitro*.

### Live-cell analysis of mitosis in HeLa cells finds that STC-treatment, but not monastrol-treatment, inhibits Eg5-mediated net depolymerization of mitotic spindle microtubules *in vivo*

We next asked whether or not the behavior of truncated Eg5 forms for study *in vitro* can extend to the native tetramer *in vivo*. We would predict, due to its ability to inhibit Eg5-mediated microtubule depolymerization, that STC-treated HeLa cells would have higher overall microtubule densities in mitotic spindles than observed in monastrol-treated cells. For our analysis, we used HeLa cells that are stably transfected with GFP-tubulin. Given that the density of labeled microtubules will correlate with their net fluorescence intensity^41^, we collected images of mitotic drug-treated HeLa cells and performed 2D and 3D analyses of microtubule density in live cells. Using fixed image capture conditions, confocal images of cells were taken after 24-hour treatment with STC or monastrol. As expected^18,19^, treatment with either drug resulted in monopolar spindle formation during mitosis.

**Figure 4:**
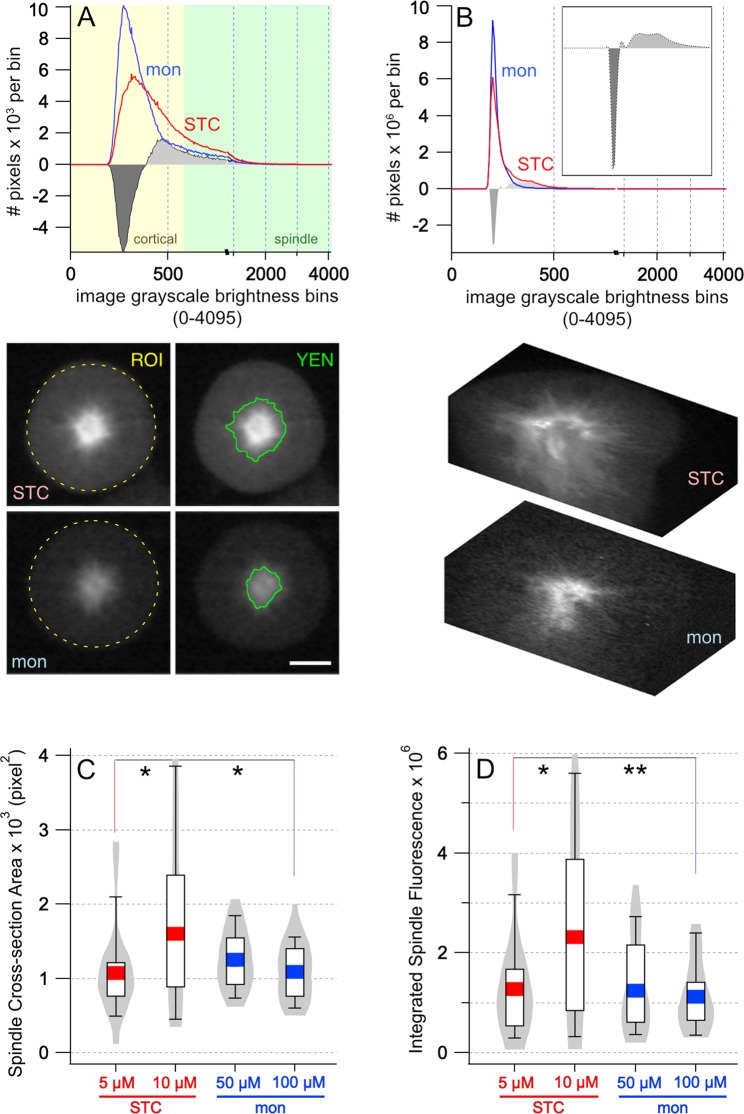
Mitotic spindle microtubule density increased with STC concentrations above the IC_50_ of Eg5 depolymerization activity. (**A**) Greyscale distribution of the cumulative fluorescence in monopolar spindles from 2D images, arising from either 100 μM monastrol (blue) or 10 μM S-trityl-L-cysteine (red) treatment of stable tubulin-GFP HeLa cells; n = 81 for monastrol-treated cells and n = 81 for STC-treated cells. Pixel intensities found in the monopolar spindles, defined by Yen cell thresholding, and in the more cortical regions of the Hela cells are highlighted by green and yellow shading, respectively. Differences in microtubule density, upon monastrol and STC treatment, were evaluated by two methods. A difference plot (grey) was created by subtraction of monastrol distribution from STC distribution. Fluorescence distributions were statistically different between monastrol and STC treated cells (D = 0.1807 > D_crit_ = 0.0651) by two-sample Kolmogorov-Smirnov measure, with greater than 95% confidence. Representative STC-treated (upper panels) and monastrol-treated (lower panels) cells, identified as close matches to the cumulative histograms, are shown; scale bar is 5 µm. Cellular fluorescence histograms were obtained using a circular region-of-interest (ROI, left panels, yellow 65 px diameter dashed circles) selection as shown on representative cell image examples. Right panels show examples of ImageJ Yen thresholding output (green contour lines) on the same cells. (**B**) Greyscale distribution of the cumulative fluorescence in monopolar spindles from z-stacks arising in stable tubulin-GFP HeLa cells treated with either 100 μM monastrol or 10 μM S-trityl-L-cysteine. After 24-hour incubation with each Eg5 inhibitor, mitotic cells exhibiting monopolar spindles were imaged by confocal microscopy and fluorescence data were collected as 200 Z-slices per cell. Cumulative distribution histograms of monastrol-treated cell tubulin fluorescence (blue) and of STC-treated cell tubulin fluorescence (red) are shown; n = 26 for monastrol-treated cells and n = 26 for STC-treated cells, which were collected from three independent experiments. Subtracting the monastrol distribution from the STC distribution results in a difference plot (in gray) that shows net positive (light grey) and net negative (dark grey) values. Two representative cells from the dataset are shown as a 3D cut-away. Box and violin plots of the (**C**) area and (**D**) total fluorescent intensity of the microtubules from stable tubulin-GFP HeLa cells were treated with 5 μM STC, 10 μM STC, 50 μM MON, or 100 μM MON for 24 hours. Results are expressed as mean (colored line) ± interquartile range in the box plot. For each condition, the Yen threshold on all the images obtained for the calculations; n = 26 from two independent cell propagations and inhibitor treatments. Outliers were removed using a Grubb’s test. P-values were determined by an unpaired two tailed t-test and values below 0.05 were considered significant (*p < 0.05; **p < 0.01).

Images of the resulting monopolar spindles were evaluated using two methods. In the first method, a fixed diameter circular region of interest (ROI) was set within each mitotic cell and a histogram showing the distribution of included pixel intensity values across all cells was calculated (Fig. 4A). The ROI sample diameter was set at the largest diameter that would fit completely within all the imaged mitotic cells; this includes the mitotic spindle and majority of the cortical regions (e.g. ROI, dashed yellow circle, left panels, Fig. 4B). With this approach, the ROI included the same total number of pixels for each cell image examined. For both monastrol- and STC-treated cell cohorts, the intensity distributions of all ROI pixels were directly summed to generate a cumulative distribution. The overall cumulative distributions integrate the effects of each drug on spindle microtubules together with cell-to-cell variations in the expression levels of tubulin-GFP and were normalized to total cells examined.

Despite cell-to-cell variation in GFP-tubulin expression, a clear shift of net microtubule density is clearly observed in cells treated with 100 µM monastrol, compared with cells treated with 10 µM STC (top panel, Fig. 4A,B). Concentrations of these allosteric inhibitors are saturating, i.e. above the IC_50_ for Eg5 ATP hydrolysis, motility, and depolymerization (*if applicable*). In examination of 2D cross sectional information, monastrol-treated cells contain fewer lighter shade pixels that are indicative of spindle microtubules (spindle, Fig. 4A) and more dark shade background pixels in the cortical regions (cortical, Fig. 4A). This is readily apparent in the difference histogram generated by subtracting the monastrol-binned data from the STC-binned data (Fig. 4A, gray). The two-sample Kolmogorov-Smirnov test confirms that data from the STC- and monastrol-treatments are distinct and do not arise from a shared distribution function. Analysis of 3D cellular volume in HeLa cells treated with monastrol or STC produced similar results. The cumulative histogram distribution over the entire Z-stack for each cell result in distinct populations of lighter shade pixels for STC-treated cells versus monastrol-treated cells (Fig. 4B). These differences in grayscale intensity were apparent in the 3D reconstruction (Fig. 4B). Thus, monopolar spindles created with STC have higher microtubule density than spindles created with monastrol.

In the second method of automating live cell image evaluation, a Yen image thresholding function^42^ was executed to go beyond the image histograms and gain a quantitative and morphological analysis of inhibitor-induced microtubule density differences. In ImageJ^43^, this function is an entropic-based algorithm to separate information-rich low entropic microtubule dense regions from high entropic background in the inhibitor-treated cells (e.g. YEN, bottom panel, Fig. 4A). In agreement with the total histogram analysis, cells treated with 100 µM monastrol displayed a significantly smaller overall area of low-entropic tubulin image information than cells treated with 10 µM STC (Fig. 4C; n_MON_ = 26, n_STC_ = 26, p = 0.0351). Additionally, we found that the monastrol-treated cells had about half the total fluorescent intensity of the microtubules in STC-treated cells (Fig. 4D; monastrol = 1,116,988, STC = 2,197,395, p = 0.0067).

However, when HeLa cells are treated with 5 µM STC, concentrations near its IC_50_ for depolymerization, the spindle cross-sectional area (Fig. 4C; p = 0.9395) and the integrated fluorescence (Fig. 4D; p = 0.5103) did not significantly differ from cells treated with 100 µM MON. No substantial deviation in area or fluorescence was observed between Hela cells treated with 50 or 100 µM monastrol (Fig. 4C,D; p = 0.1204 and p = 0.5803), the latter being near its IC_50_ for ATP hydrolysis and motility. Thus, significant *in vivo* change in microtubule density corresponded to increasing STC concentration, whereas no morphological changes to the mitotic spindle were observed as a function of monastrol concentration. Lastly, assuming that the area highlighted by the thresholding function is a circle, the radius or average microtubule length can be calculated, which was also found to be significantly shorter in 100 µM monastrol-treated cells compared to 10 µM STC-treated cells by an unpaired two-tailed t-test (data not shown).

This work on live cultured cells demonstrated STC-treated cells have more dense and longer microtubules radiating from the center of the aster compared to monastrol-treated cells. The increased microtubule-density and length in STC-treated cells correlate well with our *in vitro* Eg5 results. STC inhibits Eg5 depolymerase activity *in vivo* with dose-dependent kinetics and with an IC_50_, distinct from STC-mediated inhibition of motor ATPase activity.

## Discussion

Extensive cytoskeletal remodeling occurs in eukaryotes, both in single cell organisms and tissues. A canonical example is the formation of the mitotic spindle apparatus for chromosome segregation, during which microtubules are nucleated, bundled, and organized into a symmetrical array. Molecular motors work in concert during these cytoskeletal remodeling events to regulate the polymerization dynamics of the cytoskeleton, as well as to construct higher order structural elements. Understanding how motor proteins affect the microtubule cytoskeleton is a pre-requisite for understanding the morphogenesis of higher-order cellular machines, such as the mitotic spindle. The importance of the motility and cross-linking functions of Kinesin-5 homotetramers in mitotic spindle assembly is well established, but it is becoming increasingly clear that Kinesin-5 in human cells is capable of other functional outcomes besides motility and cross-linking microtubules.

**Figure 5:**
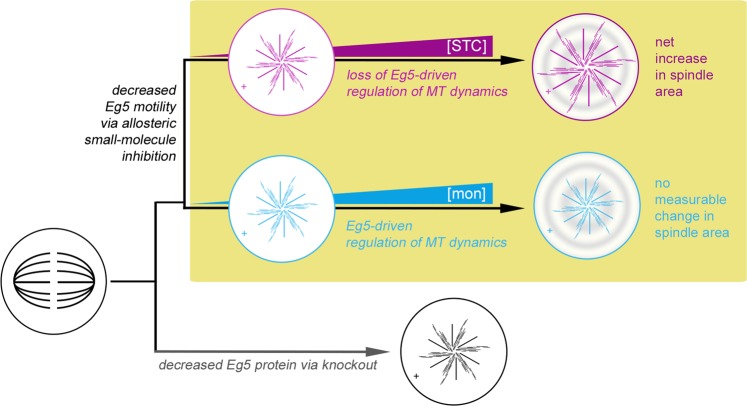
Allosteric inhibitors uncover a new role for human Kinesin-5 in the mitotic spindle that can be uncoupled from its canonical motile role in spindle assembly. Application of siRNA or other knockout methods to lower or eliminate Eg5 protein in cells give rise to a monopolar spindle phenotype (bottom row). Monastrol and STC are used ubiquitously and interchangeably in cell culture as a means to eliminate functional Eg5 during mitosis. Concentrations used in this work are above the IC_50_ values of both drugs, based on *in vitro* motility or ATPase activities. At monastrol concentrations above what is required to inhibit motor movement along the cytoskeletal track, Eg5 exhibits loss of motility, but does not lose its ability to regulate MT-dynamics (middle row). Therefore, there is no detectable change in spindle area or MT-fluorescence intensity between differing concentrations of monastrol. At STC concentrations 2X above the IC_50_ needed to inhibit depolymerization of microtubules, loss of both Eg5 motility and Eg5 regulation of MT dynamics results in net increase of spindle area and MT-fluorescence intensity (top row).

Our work illustrates a new role for Eg5 in the mitotic spindle (Fig. 5): human Kinesin-5 can modulate microtubule dynamics directly through interactions between the motor domain and microtubule filament. Thus, human Eg5 is a dual-function kinesin: it has a microtubule depolymerizing activity, in addition to its known motile functions. Although both STC and monastrol are synonymous in their allosteric inhibition of the Eg5 ATPase engine of the motor, the former exhibits robust inhibition of depolymerizing activity while the latter maintains wildtype levels of this activity. Therefore, loop5-binding inhibitors are able to selectively uncouple these two motor functions. Disparity between STC and monastrol impact on Eg5 microtubule depolymerization allowed us to look for a functional role for this novel small-molecule action in dividing cultured cells. Our data argue for a new role for human Kinesin-5 that can be uncoupled from its canonical motile role in spindle assembly. Eg5 utilizes force-production and motility in the 3D architectural assembly of the spindle, but also independently acts to regulate the length and number of individual spindle microtubules.

### Kinesin-5 is a multifunctional motor, like Kinesin-8

This dual functionality may not be unique to the human Kinesin-5. The yeast Kinesin-5 homolog, Cin8p, also exhibits a microtubule depolymerase activity (Fig. 1) and is proposed to regulate the overall length of its microtubule tracks by causing the shortening of microtubules upon reaching their plus-ends. Our conclusion is also consistent with Kinesin-5 in porcine kidney epithelial cells restricting spindle elongation, although inhibition of elongation was attributed to a brake in Eg5 motility^6^. Having a combined ability to crosslink and slide microtubules, as well as induce changes in microtubule length, allows coordination between these two functions in a single molecular entity. These data suggest that the multitasking ability of Kinesin-5 may be evolutionarily conserved.

Another family of mitotic kinesins, Kinesin-8, has been shown to have motile ability and capable of shortening the length of microtubules. The depolymerase activity of Kinesin-8 motor domain has been reported as 0.4 ± 0.3 nm/s and the longer construct of motor domain and neck linker can depolymerize Taxol-stabilized microtubules at a mean rate of 1.3 ± 0.7 nm/s^44^. The depolymerase rate of MCAK is 16 nm/s^27^. The maximum Eg5 motor domain MT depolymerization rate of 2.4 nm/s (Fig. 2D) is nearly twofold greater than Kinesin-8, but yet is less than a quarter that of MCAK. Therefore, dual-function kinesins (Kinesin-5 and Kinesin-8) have lower rates of microtubule shrinkage, compared to a single-function kinesin depolymerizer. These data suggest that the mechanotransduction energy derived from ATP hydrolysis is parsed between the dual functions of Kinesin-5 and -8 family members.

Despite the consensus between the above Kinesin-5 studies and approaches, our conclusions are on the surface at odds with recent reports: a protein constructed from a *Xenopus* Eg5 motor domain coupled to a Kinesin-1 dimerization domain was observed to promote individual microtubule elongation and polymerization *in vitro*^11,45^. However, there is some experimental accord as well: the *Xenopus* Eg5/Kinesin-1 chimera can mediate ‘shrinkage’ or depolymerization of microtubules (−79 nm/s)^11,45^, rates that exceed the ones reported here for human Eg5 and previously reported for Kinesin-13 (MCAK)^27^.

Although we do not observe net microtubule polymerization or elongation in either our *in vitro* or *in vivo* experiments, it is possible that there may be allosteric circumstances in which Kinesin-5 could favor microtubule elongation. For example, inter-species differences between kinesin isoforms have challenged the field at large: they may arise from limits in cell size that differ between organisms, physical limits in the spindle itself, or the varying number of interacting proteins between species. In addition, even within a single organism, there may be multiple members of a kinesin family: *Xenopus* has several distinct Kinesin-5 variants, whereas *Homo sapiens* only have a single Kinesin-5. Functional and cellular differences between family isoforms in a single organism are well-documented. For example, vertebrates express three Kinesin-8 proteins: (i) Kif18A mainly functions during chromosome positioning, (ii) Kif18B controls astral microtubules, and (iii) Kif19A is involved in regulation of cilia length. Although the three vertebrate Kinesin-8 proteins all display motility, reports concerning their ability to modulate microtubule dynamics vary^46,47^.

Second, experimental details make direct comparisons of *in vitro* experimental outcomes difficult. For example, *Xenopus* Kinesin-5 can exhibit microtubule stabilizing effects when tested against microtubules that are shifted to conditions that favor net catastrophe^45^. In contrast, our study revealed a human Eg5 microtubule depolymerase activity when tested against microtubules under conditions that would otherwise favor net microtubule polymerization. These discrepancies can be attributed, in part, to intrinsic differences between these Kinesin-5 homologs (*discussed above*), but also to experimental choice of microtubule stabilizing agents (Taxol, DMSO^45^, or GMPPCP^11,45^), temperature (22 °C or 37 °C)^45^, pH, etc. It is possible that the potential of Kinesin-5 to impact microtubule dynamics is to some extent subordinate to the more global variables that promote the net growth/shrinking state of the microtubule.

### Targeted inhibitors against human Kinesin-5 can be promiscuous or selective for individual Eg5 mitotic functions

Our key observation is that chemically distinct loop5-directed allosteric ATPase inhibitors can differentially control Eg5 depolymerase activity. In our assay, microtubule depolymerizing activity is strongly inhibited in the STC-bound motor domain, while monastrol treatment fails to inhibit the depolymerizing activity. This unexpected result gives rise to a number of important insights concerning the depolymerase mechanism. AMPPNP, an active site inhibitor, and STC, a loop5 pocket-directed allosteric inhibitor, both strongly inhibit ATPase activity, prevent motility, and eliminate depolymerase activity. However, while monastrol can also significantly inhibit the motor ATPase activity, it does not inhibit the microtubule depolymerase activity of the motor. Monastrol treatment appears to uncouple the motor ATPase engine and motility from microtubule depolymerizing activity. Therefore, it is likely that the persistent microtubule depolymerizing activity that we observe for the motor does not require rapid repeated cycling of the motor though its ATPase sequence.

This remarkable disparity in the outcomes of these two loop5-directed allosteric ATPase inhibitors points to the complexity of the allosteric control mechanisms that originate from loop5 of this enzyme. Future efforts will be directed at understanding how STC binding at loop5 is able to strongly inhibit the depolymerizing activity while monastrol binding does not. It is not clear how microtubule depolymerization would be favored given that the monastrol bound motor domain appears to interact with the microtubule lattice in a weakly bound state that is capable of near frictionless diffusive motion^39,48^. Nonetheless, there is evidence that kinesins can interact differently, i.e. more strongly, with the structurally distinct microtubule plus-ends along the GTP-cap^49^. It will be important to determine whether or not the monastrol-bound motor exhibits a higher affinity for the microtubule plus-ends, whereby it may stimulate depolymerization through a similar mechanism as Cin8p; a possibility that will be the focus of future experiments.

### What is the cellular role of the microtubule depolymerizing Eg5 activity?

Our data reveal that Eg5 microtubule depolymerizing activity is not required for monopolar spindle formation caused by loop5-targeting inhibitors (Fig. 5). It follows that it is also not implicated in the motile force production component of Eg5 during mitotic spindle pole separation. Yet the inability of monastrol to inhibit Eg5 microtubule depolymerizing activity may reveal a role for the native enzyme in limiting the overall microtubule density in a typical bipolar mitotic spindle. In contrast, while budding yeast Kinesin-5, Cin8p, similarly mediates microtubule disassembly, it limits its activity to kinetochore fibers present within the confines of the nuclear compartment and does not normally affect astral microtubules. However, when the Cin8p NLS is ablated, cytoplasmic microtubules are now impacted^9^. In mitotic human cells, Eg5 compartmentalization is instead mediated by post-translational modifications that regulate the association of the motor with spindle microtubules^50^. The propensity of Kinesin-5 to mediate net polymerization^11^ and/or depolymerization^9^ (and this work) may depend on crosstalk from the microtubule network itself, with the net outcome perhaps regulated by specific catastrophe frequencies at different locations in the microtubule network. Although considerable work has been done to measure spindle microtubule size distribution, half-life, and movements^51–53^ our Eg5 data indicate that not only do motor proteins control the size and shape of the mitotic spindle, but they also participate in a mechanism that regulates the overall microtubule density of the spindle. The regulation of spindle microtubule density may operate to establish an optimal ratio between polar versus kinetochore microtubules and thereby regulate opposing forces within the spindle and impact overall spindle size and net force production for mitosis.

In addition, the depolymerizing activity that we characterize in our study may point to additional cellular roles for the motor outside of its canonical function in mitotic spindle assembly. For example, recent experiments identify Eg5 as an important player in the maturation of neuronal processes, particularly dendrites, wherein the motor participates in organizing microtubule structures that are key to dendrite formation^54–56^. It is possible that the motor plays a role in establishing the polarity and stability of microtubule bundles that drive the morphological changes that characterize neuronal maturation. Future efforts will identify the cellular processes and machinery for which this additional functional activity of this kinesin play an important role.

## Materials and Methods

### Human Eg5 protein expression and purification

Cloning of the untagged. native human Kinesin-5 motor domain (residues 1–369) into pET28A for protein expression was described previously^14^. Briefly, the Eg5 motor domain was expressed in BL21 (DE3) cell lines (Invitrogen) and purified by cation exchange chromatography^23^. The Eg5 motor domain basal ATPase rates were 0.209 ± 0.030 (n = 10) and its microtubule-stimulated activity was 6.615 ± 1.098 (n = 9), consistent with literature reports^17,57–59^. The human Eg5-513 plasmid, a gift from Susan Gilbert (Rensselaer Polytechnic Institute)^33^ was transformed into BL21(DE3)RIL cells (New England Biolabs), and *E*. *coli* were cultured with ampicillin selection. Cell lysates were produced as described^33^ at 4 °C and Eg5-513 was purified by Ni-affinity chromatography using a His-Trap column and AKTA chromatography system (GE Life Sciences). Eluted protein was further purified by gel filtration using a Hi-Prep S200 column (GE Life Sciences) and buffer-exchanged into ATPase buffer (20 mM HEPES, pH 7.2 with KOH, 5 mM magnesium acetate, 0.1 mM EDTA, 0.1 mM EGTA, 50 mM potassium acetate, and 1 mM dithiothreitol) containing 0.1 mM ATP at 4 °C. The protein peak was concentrated using a Centricon centrifugal dialysis device. Glycerol (20% v/v) was added to protein fractions before snap freezing in liquid nitrogen. Using NADH-coupled activity assays, Eg5-513 basal and MT-stimulated ATPase rates were at 0.026 ± 0.002 s^−1^ (n = 43) and 0.36 ± 0.04 s^−1^ (n = 11). Experiments and procedures were approved by the institutional biosafety committee.

### Preparation of tubulin

Unlabeled tubulin was prepared essentially as described in^26^. Briefly, bovine brains were peeled and homogenized in depolymerization buffer (50 mM MES, 1 mM CaCl_2_, pH to 6.6 with NaOH) at 4 °C. After centrifugation at 80,000 × *g* at 4 °C for 60 minutes, the supernatant with same volume HMPB/ATP/GTP buffer (1 M PIPES, 10 mM MgCl_2_, 20 mM EGTA, pH to 6.9 with KOH, 4.5 mM MgATP and 1.5 mM MgGTP) and glycerol (1:1:1) to induce microtubule polymerization. Polymerization proceeded with continuous stirring at 37 °C for 1 hr. The solution was centrifuged at 160,000 × *g* for 30 min at 37 °C. The pellet was resuspended in depolymerization buffer and incubated at 4 °C to depolymerize the microtubules. Tubulin was polymerized anew and centrifuged a second time as before. The microtubule pellet was resuspended as before and subject to depolymerization at 4 °C. Subsequently the solution was clarified by centrifugation at 100,000 × *g* for 30 min at 4 °C, then the supernatant containing soluble tubulin was dispensed into 10 mg/ml aliquots and frozen in liquid N_2_. Aliquots were stored at −80 °C until immediately before use. Experiments have approval by the institutional committees for biosafety and institutional animal care and use.

### Depletion of nucleotides by alkaline phosphatase

Calf intestinal alkaline phosphatase (CIP; Sigma P7640) unit activity was calibrated to that of Eg5 motor domain to ensure comparable NTPase activity. NTPase activity was measured using the Malachite Green (MG) Phosphate Assay Kit (Bioassay Systems, Hayward, CA). We analyzed a phosphate standard curve in a 96-well plate by using a SpectraMax M2E spectrophotometer at OD_620nm_. We prepared 1 U/µl (100 mg/ml) CIP with CIP buffer (10 mM Tris-HCl, pH 8.2, 50 mM KCl, 1 mM MgCl_2_, 0.1 mM ZnCl_2_). We then prepared a dilution series of CIP at 0.1, 0.01, 0.001 and 0.0001 U/µl with TAM buffer (50 mM Tris pH 7.4, 2 mM MgCl_2_) with 100 µM ATP to final 40 µl reaction for 1 min and then added 10 µl MG. We measured OD_620nm_ at 30 min of this end-point assay and matched to the phosphate standard curve to determine the ATP hydrolysis rate. Rates were: 5.84 µM PO4/µM motor s^−1^ for MT-stimulated Eg5 hydrolysis and CIP hydrolysis rate = 7.17 µM PO_4_/U CIP s^−1^ for CIP. We note that 2.04 U CIP had equivalent hydrolysis ATP rates as 2.5 µM human Eg5 with MT.

### Microtubule depolymerization turbidity assay

Microtubule polymerization and depolymerization behaviors were measured by light scattering essentially as described^27,29^, but with the following modifications. 750 µg tubulin was polymerized in a final volume of 140 µl in a buffer containing 100 mM PIPES, pH 6.8, 2 mM EGTA, 1 mM MgCl_2_, 1 mM ATP, 1 mM GTP. The polymerization reaction was prepared at 4 °C in 96-well plate format and polymerization was initiated by incubating the reaction at 37 °C. The turbidity of the reaction was monitored at 340 nm every two minutes for up to 42 min in the temperature-controlled SpectraMax M2E spectrophotometer. Upon reaching a plateau of maximum turbidity/polymerization, 10 µl each of various effectors were added depending upon the experiment. Effectors included Eg5 motor domain (2.5 µM final concentration); monastrol (100 µM); STC (10 µM); AMPPNP (1.0 mM); CaCl_2_ (5 mM). Plate reader data was exported to Microsoft Excel and graphical illustrations, curve fitting, and histograms were generated using Igor Pro (Wavemetrics). At least three separate tubulin preparations were utilized during the course of these experiments.

### Microtubule Co-sedimentation assay

Human Eg5 protein (2 µM final) was mixed with paclitaxel-stabilized bovine brain MTs (20 µM tubulin final), and 1 mM MgATP in BRB80 buffer (80 mM Pipes, pH 6.8, 0.5 mM EGTA, and 2 mM MgCl_2_). In two separate reactions, either 100 µM monastrol or 10 µM STC were added to the above mixture. Lastly, a control sedimentation reaction of tubulin alone was used to monitor self-assembly of microtubules in the absence of chemical stabilizers. Assay reactions were incubated at 37 °C for 1 hour. Samples were centrifuged at 100,000 × *g* for 6 min at 37 °C to separate pellet (MT-bound Eg5 protein) and supernatant (soluble Eg5 protein) fractions. The pellet fractions and supernatant fractions were analyzed by SDS-PAGE.

### Total internal reflection fluorescence microscopy

A 0.24 µM mixture of AlexaFluor-555-labeled and unlabeled MTs were prepared in 1xPM (100 mM PIPES, pH 7.0, MgSO_4_, 1 mM EGTA) with 1 mM MgATP, 1 mM NaGTP, 0.4 µM Taxol, and gloxy [0.1 µg/ml glucose oxidase (Sigma), 2 µg/ml glucose catalase (Sigma)]. For TIRF measurements, 7 µl of 0.24 µM polymerized tubulin mixture was placed between a glass slide (1 mm thickness, 75 × 25 mm^2^) and plasma-cleaned coverslip (150 μm thickness, 22 × 22 mm^2^) for all measurements. For measurements with motor, a final concentration of 0.4 µM Eg5-367 or 0.2 µM Eg5-513 was used. For measurements with allosteric Eg5 inhibitor, concentrations are detailed in the figures.

Time-lapse imaging of depolymerizing labeled MTs with or without motor was performed on an Olympus IX81 inverted microscope using a 60X 1.49 NA Plan Apochromat TIRFM oil objective at room temperature. Images were acquired with a Melles Griot HeNe 543 nm laser and a Hamamatsu ORCA-ER CCD digital camera. All hardware was controlled by Micromanager software operating on the ImageJ platform.

### Live cell culture, cell lines, and confocal microscopy and analysis

Stably transfected HeLa-tubulin-eGFP cells were a gift of Tim Mitchison (Harvard Medical School). Cells were propagated according to the American Type Culture Collection protocols in medium from Gibco/Thermo/Fisher. Cells were in growth medium containing 20 mmol/L HEPES (pH 7.6) or CO_2_-independent medium (Invitrogen) with 10% fetal bovine serum, penicillin/streptomycin, and 4 mmol/L glutamine in glass-bottomed 35 mm tissue culture dishes (Delta-T dishes, Bioptechs). Cells were treated with media containing either 50 or 100 µM monastrol and 5 or 10 µM STC for 24 hrs before imaging. Cells were imaged on a Zeiss Axiovert 200 equipped with Andor/Yokogawa spinning disk confocal system and Hamamatsu Orca-ER camera controlled by μManager software^60^. Cells were maintained at 37 °C while imaging by a Bioptechs plate warming system and microscope objective warmer. A Bioptechs heated lid was fitted to the culture dishes and a CO_2_ stream was used to maintain media pH during the experiments. Cells were imaged through a Zeiss 63X NA 1.4 DIC lens.

Drug-treated HeLa cells were examined after 24 hrs drug treatment, and tubulin-channel images were collected from all mitotic cells. Image capture conditions and settings were held constant throughout the experiment. After image collection, any images displaying pixel intensity saturation were rejected from subsequent analysis. Also, images wherein the mitotic cells exhibited morphological abnormalities, such as non-round shape or apoptosis features, were also rejected from analysis. Morphologically normal cells exhibiting monopolar spindles were selected for analysis. After this quality control step, 26 STC-treated cells and 26 monastrol-treated cells across two independent experiments were analyzed. Images were processed using ImageJ software^61^.

For 2D analysis, all the selected mitotic HeLa cells were spherical and approximately 72 pixels in diameter in our images; therefore, a circular ROI of 65 pixels in diameter was used to capture images from each mitotic cell. Image pixel intensity/grey level histogram data were extracted from each ROI and exported as comma-separated files (.csv) for analysis in Microsoft Excel. Statistical significance of the pixel intensity distribution plots was determined using the two-sample Kolmogorov-Smirnov test. For 3D analysis, a circular ROI of 100 pixels in diameter was used to capture the entire cell, through 200 slices from each stack at 0.2 µm step per slice. The grey-level histogram of each slice was used as a direct readout of tubulin polymer without normalization or the application of thresholding algorithms. The gray level histogram data encompassing the entire stack was extracted using ImageJ. A total of 26 cells were analyzed and averaged for each condition, over three independent experiments. The 3D cut-away was generated using MATLAB (Mathworks).

Alternatively, ImageJ built-in Yen image thresholding algorithm was used to evaluate spatial fluorescence localization and density in 2D cross-sectional images. The Yen algorithm exploits the entropy of the distribution of gray levels in an image and determines the boundary of foreground information from background; the algorithm returns a binary thresholded image; an area of maximum information entropy vs an area of noise^42^. Statistical significance of the thresholded 2D data was established by a combination of Grubb’s test to remove outliers. By two-way ANOVA, there was a significant interaction between the effects of concentration and drug on both the overall intensity (F(1,100) = 5.21, p = 0.0245) and area (F(1,100) = 6.36, p = 0.0132) of the microtubule spindle. Simple main effects analysis showed that there is a significant increase in microtubule area (p = 0.0440) and fluorescent intensity (p = 0.0260) between 5 μM and 10 μM STC and between 10 μM STC and 100 μM MON (area, p = 0.0351 and fluorescent intensity, p = 0.0067). These statistical results are shown in Fig. 4.

Graphical illustrations and histograms were generated using Igor Pro (Wavemetrics). Means and interquartile range are shown in the box plots. Quartile calculation method was Tukey; whiskers feature the 9^th^ and 91th percentile. Violin plots were computed using the Silverman bandwidth method, an Epanechnikov kernel, data jitter of 0.5, and a curve extension of 0.1, and default distribution maximum of 4.8e-07.

## Supplementary information

Kim et al Supplement SREP Eg5 Depolymerase

## Data Availability

All data generated or analyzed during this study are included in this published article.
