# Supplementary material for: Small molecule allosteric uncoupling of microtubule depolymerase activity from motility in human Kinesin-5 during mitotic spindle assembly
Source: Sci Rep. 2019 Dec 27;9:19900. doi: 10.1038/s41598-019-56173-9 (PMC6934681; doi:10.1038/s41598-019-56173-9)
Supplement: Supplementary file 1 — Kim et al Supplement SREP Eg5 Depolymerase [file 41598_2019_56173_MOESM1_ESM.pdf]

SUPPLEMENT

# Small molecule allosteric uncoupling of microtubule depolymerase activity from motility in human Kinesin-5 during mitotic spindle assembly

Catherine D. Kim, Elizabeth D. Kim, Liqiong Liu, Rebecca Buckley,
Sreeja Parameswaran, Sunyoung Kim, and Edward J. Wojcik

## Affiliations

Department of Biochemistry and Molecular Biology,
LSU School of Medicine & Health Sciences Center,
New Orleans, LA 70112

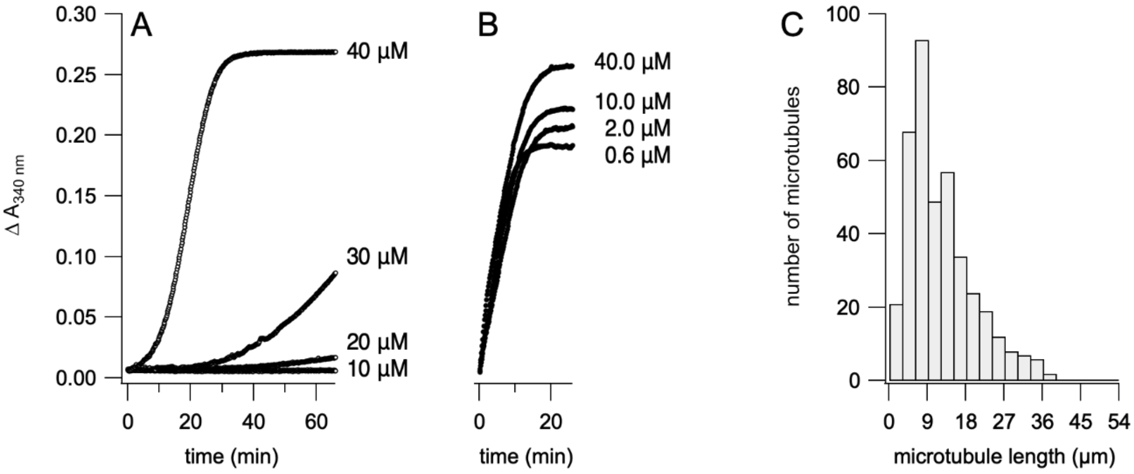


**Figure S1. Tubulin can assemble *in vitro* with and without chemical stabilizers. (A)** Critical tubulin concentration for *in vitro* self-assembly is 40 micromolar in the absence of chemical microtubule stabilizers. Four different concentrations of purified, bovine tubulin were incubated at 37$^{o}$C in the presence of 1X PM, 1 mM GTP, 1 mM ATP, and 3% DMSO. ***(B)*** Microtubules can form with less than 1 $\mu$M tubulin in the presence of 20 $\mu$M Taxol. Tubulin concentrations are shown in the panel. ***(C)*** The median microtubule length formed *in vitro* is 10.1 ± 7.7 (SD) $\mu$m. Histogram shows the length distribution of microtubules formed. An aliquot of 40 $\mu$M unlabeled and AlexaFluor-555-labeled tubulin was mixed with an equimolar amount of Taxol; the reaction mixture was incubated at 37 $^{o}$C for 1 hour to permit polymerization. The chemically stabilized microtubules were diluted 200X in 1X PM, 1 mM GTP, 1 mM ATP, 0.4 $\mu$M Taxol, and 2 $\mu$g/ml catalase and 0.1 $\mu$g/ml glucose oxidase. A 7 $\mu$L aliquot was sandwiched between with coverslips and used to experimentally determine microtubule length by TIRF. Five independent rounds of microtubules were assembled; a single aliquot from each tubulin polymerization round was used for TIRF measurement; N=400 microtubules.

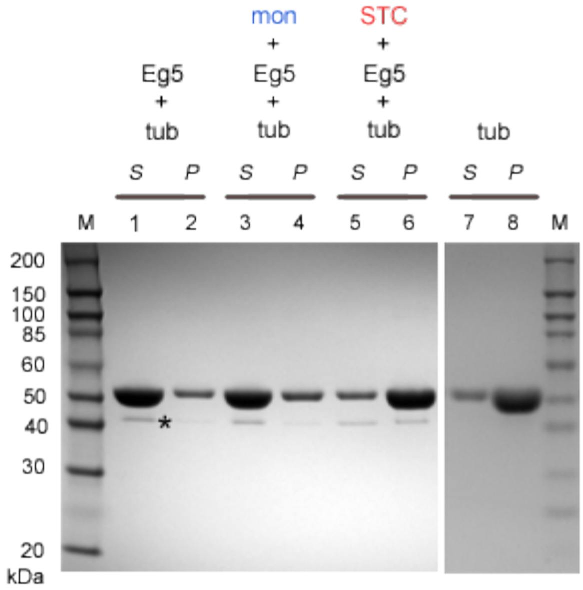


**Figure S2. Microtubule pull-down assay reveals net microtubule depolymerization by monastrol-treated Eg5 and similar reduced affinity to microtubules as STC-treated motor.** SDS-PAGE of standard microtubule pull-down assays[55] were loaded with either pellet (P) or supernatant (S) fractions of each condition. Each reaction contained GTP-microtubules (upper band in each lane), Eg5 motor domain (marked with *), and 1 mM ATP. Sample preparations were treated with either vehicle (DMSO), 10 μM S-trityl-L-cysteine (STC), or 100 μM monastrol (MON). Both monastrol and STC-treated Eg5 motor domain had lower association with microtubules (lanes 4 and 6, respectively). Note the lower amount of pelleted tubulin in the monastrol treated pellet fraction (lane 4), similar to that of Eg5 alone (lane 2), as compared with the STC-treated fraction (wherein Eg5-mediated microtubule depolymerization is inhibited, lane 6). Control tubulin was run in a replicate experiment and is shown in lanes 7 and 8. Abbreviations: Eg5, human kinesin-5; tub, tubulin; mon, monastrol; STC, S-trityl-L-cysteine; M, molecular weight ladder.
